# Supplementary material for: Quantitative prediction of grain boundary thermal conductivities from local atomic environments
Source: Nat Commun. 2020 Apr 15;11:1854. doi: 10.1038/s41467-020-15619-9 (PMC7160158; doi:10.1038/s41467-020-15619-9)
Supplement: Supplementary file 1 — Supplementary Information [file 41467_2020_15619_MOESM1_ESM.pdf]

Supplementary Information for

Quantitative prediction of grain boundary thermal conductivities from local atomic environments

Susumu Fujii[1,2,3], Tatsuya Yokoi[3,4], Craig. A. J. Fisher[1],

Hiroki Moriwake[1,2], Masato Yoshiya[1,3,5]

[1] Nanostructures Research Laboratory, Japan Fine Ceramics Center, Nagoya 456-8587, Japan

[2] Center for Materials Research by Information Integration, National Institute for Materials Science, Tsukuba 305-0047, Japan

[3] Department of Adaptive Machine Systems, Osaka University, Osaka 565-0871, Japan

[4] Department of Materials Physics, Nagoya University, Nagoya 464-8603, Japan[3]

[5] Division of Materials and Manufacturing Science, Osaka University, Osaka 565-0871, Japan

Supplementary Table 1 List of symmetric tilt grain boundaries (STGBs) with [001] rotation axis.

Grain boundary, GB	GB type	Tilt angle, 2θ (°)	GB energy, E^{GB} (Jm^{-2})	Excess volume, V^{GB} (Å)	Effective thermal conductivity, κ_{eff} ($Wm^{-1}K^{-1}$)
Σ221(21 1 0)/[001]	low-angle (open)	5.45	1.162	0.433	30.79 ± 0.91
Σ113(15 1 0)/[001]	low-angle (open)	7.63	1.374	0.582	22.55 ± 0.69
Σ101(10 1 0)/[001]	low-angle (open)	11.42	1.633	0.796	17.21 ± 0.51
Σ41(910)/[001]	low-angle (open)	12.68	1.696	0.858	17.46 ± 0.45
Σ65(810)/[001]	low-angle (open)	14.25	1.763	0.916	15.61 ± 0.49
Σ25(710)/[001]	high-angle	16.26	1.826	0.911	13.79 ± 0.42
Σ37(610)/[001]	high-angle	18.92	1.888	1.012	12.44 ± 0.49
Σ13(510)/[001]	high-angle	22.62	1.951	1.137	10.79 ± 0.58
Σ17(410)/[001]	high-angle	28.07	1.987	1.130	11.83 ± 0.49
Σ53(720)/[001]	high-angle	31.89	2.020	1.228	11.31 ± 0.59
Σ5(310)/[001]	high-angle	36.87	1.947	1.235	11.24 ± 0.64
Σ29(520)/[001]	high-angle	43.60	2.015	1.175	11.55 ± 0.44
Σ5(210)/[001]	high-angle	53.13	1.761	0.994	14.07 ± 0.63
Σ13(320)/[001]	low-angle (dense)	67.38	2.055	0.388	19.37 ± 0.80
Σ25(430)/[001]	low-angle (dense)	73.74	1.769	0.319	23.56 ± 0.84
Σ41(540)/[001]	low-angle (dense)	77.32	1.560	0.276	27.48 ± 1.03

Notes:

(1) Grey background indicates GB was used as test data.

(2) A tilt angle of 90° about the [001] axis produces a single crystal. GBs whose tilt angles are close to 0° and 90° are low-angle GBs with respect to (100) and (110) planes, respectively.

(3) Uncertainties in effective thermal conductivity values correspond to standard deviations in thermal conductivity calculated using perturbations of different magnitudes.

Supplementary Table 2 List of symmetric tilt grain boundaries (STGBs) with $[1\bar{1}0]$ rotation axis.

Grain boundary, GB	GB type	Tilt angle, 2θ (°)	GB energy, E^{GB} (Jm^{-2})	Excess volume, V^{GB} (Å)	Effective thermal conductivity, κ_{eff} ($Wm^{-1}K^{-1}$)
Σ129(881)/$[1\bar{1}0]$	low-angle (dense)	10.10	1.818	0.212	25.46 ± 0.48
Σ51(551)/$[1\bar{1}0]$	low-angle (dense)	16.10	2.284	0.303	20.46 ± 0.65
Σ51(772)/$[1\bar{1}0]$	low-angle (dense)	22.84	2.659	0.381	17.52 ± 0.89
Σ19(331)/$[1\bar{1}0]$	high-angle	26.53	2.755	0.419	16.54 ± 0.52
Σ9(221)/$[1\bar{1}0]$	high-angle	38.94	2.744	0.662	14.70 ± 0.87
Σ41(443)/$[1\bar{1}0]$	twin-like	55.88	2.564	0.362	17.22 ± 0.49
Σ33(554)/$[1\bar{1}0]$	twin-like	58.99	2.424	0.321	17.78 ± 1.02
Σ3(111)/$[1\bar{1}0]$	twin	70.53	0.862	0.009	24.83 ± 1.21
Σ57(445)/$[1\bar{1}0]$	twin-like	82.95	2.398	0.281	18.60 ± 0.74
Σ17(334)/$[1\bar{1}0]$	high-angle	86.63	2.536	0.411	17.77 ± 0.77
Σ17(223)/$[1\bar{1}0]$	high-angle	93.37	2.698	0.433	16.00 ± 0.52
Σ3(112)/$[1\bar{1}0]$	high-angle	109.47	2.545	0.911	13.94 ± 0.45
Σ11(113)/$[1\bar{1}0]$	high-angle	129.52	3.145	0.955	9.54 ± 0.43
Σ9(114)/$[1\bar{1}0]$	high-angle	141.06	2.817	1.323	9.19 ± 0.56
Σ27(115)/$[1\bar{1}0]$	high-angle	148.41	2.748	1.338	9.60 ± 0.40
Σ19(116)/$[1\bar{1}0]$	high-angle	153.47	2.528	1.263	10.08 ± 0.34
Σ51(117)/$[1\bar{1}0]$	low-angle (open)	157.16	2.444	1.209	10.12 ± 0.98
Σ33(118)/$[1\bar{1}0]$	low-angle (open)	159.95	2.307	1.107	11.00 ± 0.66
Σ51(1 1 10)/$[1\bar{1}0]$	low-angle (open)	163.90	2.143	0.999	12.89 ± 0.62
Σ171(1 1 13)/$[1\bar{1}0]$	low-angle (open)	167.58	1.938	0.858	15.04 ± 0.60

Notes:

(1) Grey background indicates GB was used as test data.

(2) GBs whose tilt angles are close to 0° and 180° are low-angle GBs with respect to (110) and (001) planes, respectively, because a tilt angle of 180° about the $[1\bar{1}0]$ axis produces a single crystal.

(3) Σ3(111)/$[1\bar{1}0]$ is a twin boundary.

(4) Uncertainties in effective thermal conductivity values correspond to standard deviations in thermal conductivity calculated using perturbations of different magnitudes.

Supplementary Table 3 List of symmetric tilt grain boundaries (STGBs) with [111] rotation axis.

Grain boundary, GB	GB type	Tilt angle, 2θ (°)	GB energy, E^{GB} (Jm^{-2})	Excess volume, V^{GB} (Å)	Effective thermal conductivity, κ_{eff} ($Wm^{-1}K^{-1}$)
Σ183(13 $\overline{14}$ 1)/[111]	low-angle (dense)	7.34	1.384	0.185	33.37 ± 1.01
Σ327(17 $\overline{19}$ 2)/[111]	low-angle (dense)	10.99	1.770	0.254	26.83 ± 0.79
Σ43(6$\bar{7}$1)/[111]	low-angle (dense)	15.18	2.142	0.319	22.60 ± 1.12
Σ147(11 $\overline{13}$ 2)/[111]	low-angle (dense)	16.43	2.215	0.328	21.84 ± 0.74
Σ31(5$\bar{6}$1)/[111]	low-angle (dense)	17.90	2.301	0.344	21.39 ± 0.71
Σ21(4$\bar{5}$1)/[111]	low-angle (dense)	21.79	2.530	0.393	19.91 ± 0.61
Σ39(5$\bar{7}$2)/[111]	high-angle	32.20	2.985	0.577	17.18 ± 0.60
Σ201(11 $\overline{16}$ 5)/[111]	high-angle	35.57	3.007	0.544	16.34 ± 0.57
Σ7(2 $\bar{3}$ 1)/[111]	high-angle	38.21	2.999	0.443	15.69 ± 0.67
Σ93(7 $\overline{11}$ 4)/[111]	high-angle	42.10	3.128	0.550	15.51 ± 0.45
Σ129(8 $\overline{13}$ 5)/[111]	high-angle	44.82	3.133	0.943	13.49 ± 0.67
Σ19(3$\bar{5}$2)/[111]	high-angle	46.83	3.106	0.652	14.27 ± 0.48
Σ3(1$\bar{2}$1)/[111]	high-angle	60.00	2.539	0.911	13.87 ± 0.48

Notes:

(1) Grey background indicates GB was used as test data.

(2) Only tilt angles from 0° to 60° need to be considered in the case of GBs formed by rotation about the [111] axis owing to the high-symmetry of the rock-salt structure.

(3) GBs whose tilt angles are close to 0° are low-angle GBs with respect to the $(1\bar{1}0)$ plane.

(4) Uncertainties in effective thermal conductivity values correspond to standard deviations in thermal conductivity calculated using perturbations of different magnitudes.

Supplementary Table 4 List of symmetric tilt grain boundaries (STGBs) with [112] rotation axis.

Grain boundary, GB	GB type	Tilt angle, 2θ (°)	GB energy, E^{GB} (Jm^{-2})	Excess volume, V^{GB} (Å)	Effective thermal conductivity, κ_{eff} ($Wm^{-1}K^{-1}$)
$\Sigma291(11\ \overline{13}\ 1)/[112]$	low-angle (dense)	11.66	1.818	0.258	24.60 ± 0.68
$\Sigma165(8\ \overline{10}\ 1)/[112]$	low-angle (dense)	15.50	2.138	0.310	21.10 ± 0.64
$\Sigma75(5\bar{7}1)/[112]$	low-angle (dense)	23.07	2.617	0.398	17.59 ± 0.55
$\Sigma87(7\ \overline{11}\ 1)/[112]$	high-angle	30.45	3.039	0.465	15.68 ± 0.35
$\Sigma105(5\ \overline{13}\ 4)/[112]$	high-angle	57.12	3.057	1.356	11.21 ± 0.48
$\Sigma11(1\bar{3}1)/[112]$	high-angle	62.96	3.145	0.955	9.54 ± 0.43
$\Sigma15(1\bar{5}2)/[112]$	high-angle	78.46	2.734	1.198	11.64 ± 0.46
$\Sigma5(0\bar{2}1)/[112]$	high-angle	101.54	1.763	0.994	14.16 ± 0.40
$\Sigma33(\bar{1}\bar{7}4)/[112]$	high-angle	117.04	2.953	0.601	14.69 ± 0.55
$\Sigma35(\bar{1}\bar{5}3)/[112]$	high-angle	122.88	3.027	0.720	13.05 ± 0.45
$\Sigma93(\bar{2}\bar{8}5)/[112]$	high-angle	127.80	3.198	0.660	14.97 ± 0.43
$\Sigma7(\bar{1}\bar{3}2)/[112]$	high-angle	135.58	3.015	0.464	16.24 ± 0.55
$\Sigma165(\bar{4}\ \overline{10}\ 7)/[112]$	high-angle	141.43	3.082	0.514	15.21 ± 0.48
$\Sigma105(\bar{5}\ \overline{11}\ 8)/[112]$	high-angle	145.95	3.006	0.570	15.32 ± 0.67
$\Sigma29(\bar{2}\bar{4}3)/[112]$	high-angle	149.55	2.825	0.607	15.19 ± 0.42
$\Sigma295(\overline{15}\ \overline{13}\ 14)/[112]$	twin-like	166.69	2.415	0.241	18.87 ± 0.34
$\Sigma3(\bar{1}\bar{1}1)/[112]$	twin	180.00	0.862	0.009	24.83 ± 1.21

Notes:

(1) Grey background indicates GB was used as test data.

(2) $\Sigma3(\bar{1}\bar{1}1)/[112]$ is a twin boundary.

(3) GBs whose tilt angles are close to 0° are low-angle GBs with respect to the $(1\bar{1}0)$ plane.

(4) Uncertainties in effective thermal conductivity values correspond to standard deviations in thermal conductivity calculated using perturbations of different magnitudes.

Supplementary Table 5 List of symmetric tilt grain boundaries (STGBs) with $[2\bar{1}0]$ rotation axis.

Grain boundary, GB	GB type	Tilt angle, 2θ (°)	GB energy, E^{GB} (Jm^{-2})	Excess volume, V^{GB} (Å)	Effective thermal conductivity, κ_{eff} ($Wm^{-1}K^{-1}$)
Σ5(120)/[2$\bar{1}$0]	high-angle	0.00	1.761	0.994	14.07 ± 0.63
Σ215(9 18 5)/[2$\bar{1}$0]	high-angle	27.91	2.807	1.071	13.28 ± 0.48
Σ135(7 14 5)/[2$\bar{1}$0]	high-angle	35.43	2.867	1.027	13.32 ± 0.51
Σ205(6 12 5)/[2$\bar{1}$0]	high-angle	40.88	2.868	0.990	13.36 ± 0.53
Σ3(121)/[2$\bar{1}$0]	high-angle	48.19	2.539	0.911	13.87 ± 0.48
Σ105(485)/[2$\bar{1}$0]	high-angle	58.41	2.873	0.660	14.68 ± 0.61
Σ29(243)/[2$\bar{1}$0]	high-angle	67.71	2.825	0.607	15.19 ± 0.42
Σ35(365)/[2$\bar{1}$0]	high-angle	73.40	2.763	0.624	14.97 ± 0.40
Σ9(122)/[2$\bar{1}$0]	high-angle	83.62	2.741	0.662	13.96 ± 0.66
Σ7(123)/[2$\bar{1}$0]	high-angle	106.60	2.999	0.443	15.69 ± 0.67
Σ145(3 6 10)/[2$\bar{1}$0]	high-angle	112.29	3.186	0.675	15.05 ± 0.54
Σ15(125)/[2$\bar{1}$0]	high-angle	131.81	2.733	1.198	11.39 ± 0.53
Σ105(1 2 10)/[2$\bar{1}$0]	low-angle (open)	154.79	2.360	1.169	10.96 ± 0.32
Σ115(1 2 15)/[2$\bar{1}$0]	low-angle (open)	163.04	2.108	1.000	12.98 ± 0.64
Σ315(1 2 25)/[2$\bar{1}$0]	low-angle (open)	169.78	1.726	0.769	17.74 ± 0.48

Notes:

(1) Grey background indicates GB was used as test data.

(2) To construct crystallographically more complex STGB structures, two half-crystals were rotated by 2θ = 53.13° about the [001] axis before being rotated about the $[2\bar{1}0]$ axis, i.e., the STGBs have two different rotation axes. Thus the GB listed as having 2θ = 0° is not a single crystal but a $\Sigma5(120)/[2\bar{1}0]$ GB, which is identical to a Σ5(210)/[001] GB.

(3) GBs whose tilt angles are close to 180° are low-angle GBs with respect to the (001) plane, because A tilt angle of 180° produces a single crystal owing to the symmetry of the rock-salt structure.

(4) Uncertainties in effective thermal conductivity values correspond to standard deviations in thermal conductivity calculated using perturbations of different magnitudes.

Supplementary Table 6 List of symmetric tilt grain boundaries (STGBs) with $[3\bar{1}0]$ rotation axis.

Grain boundary, GB	GB type	Tilt angle, 2θ (°)	GB energy, E^{GB} (Jm^{-2})	Excess volume, V^{GB} (Å)	Effective thermal conductivity, κ_{eff} ($Wm^{-1}K^{-1}$)
Σ5(130)/$[3\bar{1}0]$	high-angle	0.00	1.947	1.235	11.24 ± 0.64
Σ665(8 24 5)/$[3\bar{1}0]$	high-angle	22.36	2.780	1.278	10.90 ± 0.48
Σ385(6 18 5)/$[3\bar{1}0]$	high-angle	29.53	3.035	1.468	10.63 ± 0.46
Σ11(1 3 1)/$[3\bar{1}0]$	high-angle	35.10	3.145	0.955	9.54 ± 0.43
Σ185(4 12 5)/$[3\bar{1}0]$	high-angle	43.14	3.002	1.172	11.76 ± 0.64
Σ295(7 21 10)/$[3\bar{1}0]$	high-angle	48.62	2.892	1.081	12.78 ± 0.66
Σ7(132)/$[3\bar{1}0]$	high-angle	64.62	3.002	0.441	15.95 ± 0.45
Σ19(133)/$[3\bar{1}0]$	high-angle	86.98	2.755	0.419	16.54 ± 0.52
Σ95(3 9 10)/$[3\bar{1}0]$	high-angle	93.02	2.902	0.472	16.40 ± 0.66
Σ385(4 12 15)/$[3\bar{1}0]$	high-angle	99.72	2.951	0.402	16.65 ± 0.49
Σ35(135)/$[3\bar{1}0]$	high-angle	115.38	3.027	0.720	13.05 ± 0.45
Σ235(1 3 15)/$[3\bar{1}0]$	low-angle (open)	156.19	2.263	1.111	11.15 ± 0.42
Σ205(1 3 20)/$[3\bar{1}0]$	low-angle (open)	162.03	2.094	1.040	12.92 ± 0.55
Σ635(1 3 25)/$[3\bar{1}0]$	low-angle (open)	165.58	1.949	0.927	14.27 ± 0.78
Σ455(1 3 30)/$[3\bar{1}0]$	low-angle (open)	167.97	1.832	0.837	16.31 ± 0.58
Σ1235(1 2 35)/$[3\bar{1}0]$	low-angle (open)	169.67	1.705	0.749	18.23 ± 0.31

Notes:

(1) Grey background indicates GB was used as test data.

(2) To construct crystallographically more complex STGB structures, two half-crystals were rotated by 2θ = 36.87° about the [001] axis before being rotated about the $[3\bar{1}0]$ axis, i.e., the STGBs have two different rotation axes. Thus the GB listed as having 2θ = 0° about the $[3\bar{1}0]$ axis is not a single crystal but a Σ5(130)/$[3\bar{1}0]$ GB, which is identical to a Σ5(310)/[001] GB.

(3) GBs whose tilt angles are close to 180° are low-angle GBs with respect to the (001) plane, because a tilt angle of 180° produces a single crystal owing to the symmetry of the rock-salt structure.

(4) Uncertainties in effective thermal conductivity values correspond to standard deviations in thermal conductivity calculated using perturbations of different magnitudes.

Supplementary Table 7 List of symmetric twist grain boundaries (GBs) with [001] rotation axis.

Grain boundary, GB	GB type	Twist angle, θ (°)	GB energy, E^{GB} (Jm^{-2})	Excess volume, V^{GB} (Å)	Effective thermal conductivity, κ_{eff} ($Wm^{-1}K^{-1}$)
Σ41(001)	twist	12.68	1.924	0.173	25.90 ± 0.90
Σ25(001)	twist	16.26	2.193	0.184	18.07 ± 0.68
Σ37(001)	twist	18.92	2.320	0.268	16.13 ± 0.65

Supplementary Table 8 List of symmetric tilt grain boundaries (STGBs) with [001] rotation axis under high-pressure.

Grain boundary, GB	GB type	Tilt angle 2θ (°)	GB energy E^{GB} (Jm^{-2})	Excess volume V^{GB} (Å)	Effective thermal conductivity κ_{eff} ($Wm^{-1}K^{-1}$)
Σ25(710)/[001]	high-pressure	16.26	2.192	0.559	17.59 ± 0.76
Σ13(510)/[001]	high-pressure	22.62	2.430	0.590	14.95 ± 0.48
Σ17(410)/[001]	high-pressure	28.07	2.490	0.686	11.43 ± 0.54
Σ5(310)/[001]	high-pressure	36.87	2.335	0.644	14.50 ± 0.67

Supplementary Table 9 List of asymmetric tilt grain boundaries (GBs) with [001] rotation axis.

Grain boundary, GB	GB type	Tilt angle, θ (°)	GB energy, E^{GB} (Jm^{-2})	Excess volume, V^{GB} (Å)	Effective thermal conductivity, κ_{eff} ($Wm^{-1}K^{-1}$)
Σ5(430)(100)/[001]	asymmetric	36.87 / 0.00	2.049	1.192	13.19 ± 0.54
Σ5(740)($\bar{8}$10)/[001]	asymmetric	29.74 / 7.125	2.047	1.215	12.62 ± 0.56
Σ5(110)(710)/[001]	asymmetric	45.00 / 6.340	2.024	1.136	14.01 ± 0.55
Σ5(670)(920)/[001]	asymmetric	40.60 / 12.53	1.985	1.100	14.05 ± 0.31

Notes for Supplementary Tables 7 to 9:

(1) Grey background indicates GB was used as test data.

(2) Uncertainties in effective thermal conductivity values correspond to standard deviations in thermal conductivity calculated using perturbations of different magnitudes.

Supplementary Note 1

Supplementary Tables 1 to 9 list all GBs investigated in this study. The STGBs under standard pressure were the same as those examined in our previous study [1]. The total number of GBs examined was 108, but some of the GBs are duplicates because of the high-symmetry of the cubic rock-salt structure. The duplicate GBs are as follows:

(1) $\Sigma 3(111)/[1\bar{1}0]$ and $\Sigma 3(\bar{1}\bar{1}1)/[112]$
(2) $\Sigma 3(112)/[1\bar{1}0]$, $\Sigma 3(1\bar{2}1)/[111]$ and $\Sigma 3(121)/[2\bar{1}0]$.
(3) $\Sigma 5(210)/[001]$, $\Sigma 5(0\bar{2}1)/[112]$ and $\Sigma 5(120)/[2\bar{1}0]$
(4) $\Sigma 5(310)/[001]$ and $\Sigma 5(130)/[3\bar{1}0]$
(5) $\Sigma 7(2\bar{3}1)/[111]$, $\Sigma 7(\bar{1}\bar{3}2)/[112]$, $\Sigma 7(123)/[2\bar{1}0]$ and $\Sigma 7(132)/[3\bar{1}0]$
(6) $\Sigma 9(221)/[1\bar{1}0]$ and $\Sigma 9(122)/[2\bar{1}0]$
(7) $\Sigma 11(113)/[1\bar{1}0]$, $\Sigma 11(1\bar{3}1)/[112]$ and $\Sigma 11(131)/[3\bar{1}0]$
(8) $\Sigma 15(1\bar{5}2)/[112]$ and $\Sigma 15(125)/[2\bar{1}0]$
(9) $\Sigma 19(331)/[1\bar{1}0]$ and $\Sigma 19(133)/[3\bar{1}0]$
(10) $\Sigma 29(\bar{2}\bar{4}3)/[112]$ and $\Sigma 29(243)/[2\bar{1}0]$
(11) $\Sigma 35(\bar{1}\bar{5}3)/[112]$ and $\Sigma 35(135)/[3\bar{1}0]$

The total number of unique GBs examined is thus 92. These GB models are available as Supplementary Data 1 in LAMMPS format. GB energy, ΔE^{GB}, was calculated using the following equation:

$$\Delta E^{\mathrm{GB}} = \frac{E^{\mathrm{GB}} - \frac{N^{\mathrm{GB}}}{N^{\mathrm{SC}}} E^{\mathrm{SC}}}{2A}, \qquad (1)$$

where E^{GB} and E^{SC} are the lattice energies of the GB model and unit cell, respectively, N_{GB} and N_{SC} are the number of atoms in the GB model and unit cell, respectively, and A is the GB cross-sectional area.

We defined a low-angle GB as being an STGB whose misorientation angle is between 0° and 15° with respect to a single crystal or an STGB that can be described as an array of dislocations. A twin-like GB was defined as an STGB whose misorientation angle is between 0° and 15° with respect to the Σ3(111) twin boundary. These GBs partially consist of regions of LAEs that are similar to those in the twin boundary. Our GB dataset contained four twin-like boundaries, namely $\Sigma 41(443)/[1\bar{1}0]$, $\Sigma 33(554)/[1\bar{1}0]$, $\Sigma 57(445)/[1\bar{1}0]$ and $\Sigma 295(\overline{15}\,\overline{13}\,14)/[112]$ GBs, and these structures are illustrated in Supplementary Fig. 1 along with that of the twin boundary. $\Sigma 33(554)/[1\bar{1}0]$ and $\Sigma 295(\overline{15}\,\overline{13}\,14)/[112]$ GBs contain sections of coherently bonded, fully coordinated atoms at the boundaries like the twin. In contrast, $\Sigma 41(443)/[1\bar{1}0]$ and $\Sigma 57(445)/[1\bar{1}0]$ GBs do not contain such sections, but form another kind of symmetric structure in which the twin configurations have slid slightly along the GB plane. Because of their similarity to the twin structure, LAEs of these boundaries have relatively low LDFs compared with conventional high-angle GBs (Fig. 3b), resulting in different thermal conduction behaviour compared to high- and low-angle GBs with no twin-like sections, as seen in Fig. 1a.

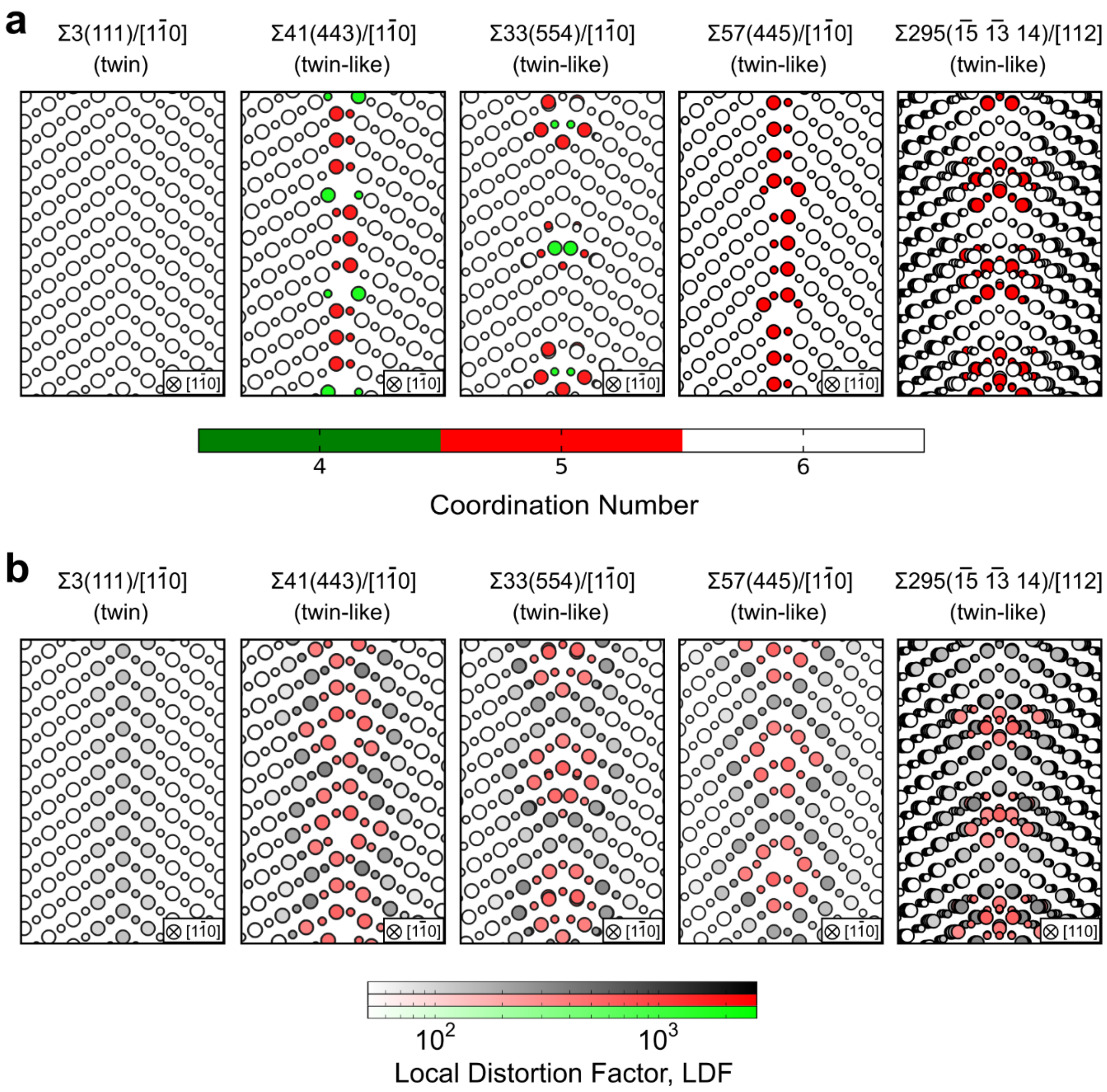

Supplementary Fig. 1 Atomic structures of twin and twin-like GBs coloured according to **(a)** their coordination number and **(b)** LAE group and LDF. Large and small balls represent Mg and O atoms, respectively. The ideal coordination numbers of Mg and O atoms are both six in rocksalt MgO.

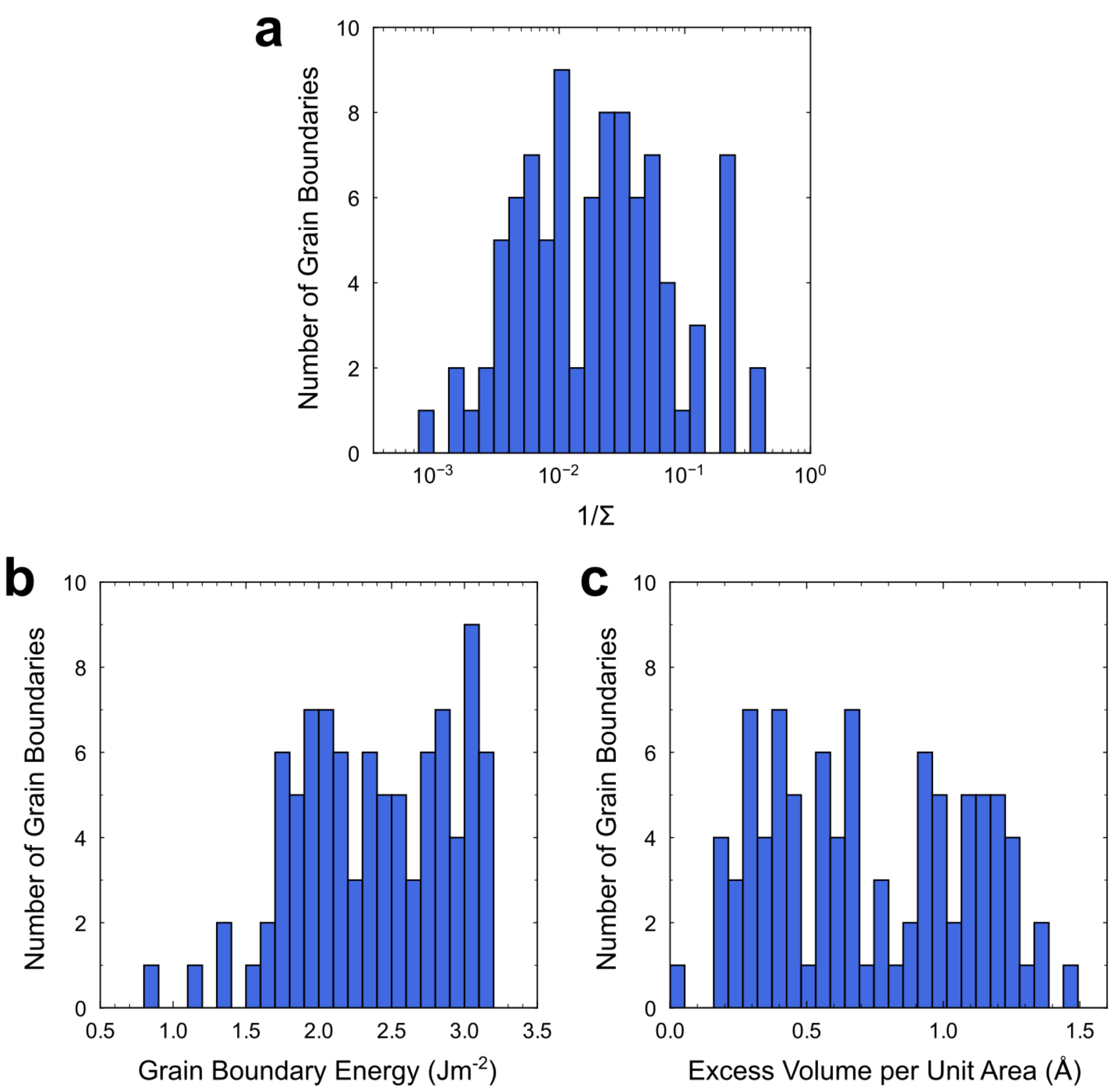

Supplementary Fig. 2 Number of grain boundaries as a function of **(a)** 1/Σ, **(b)** grain boundary energy, and **(c)** grain boundary excess volume.

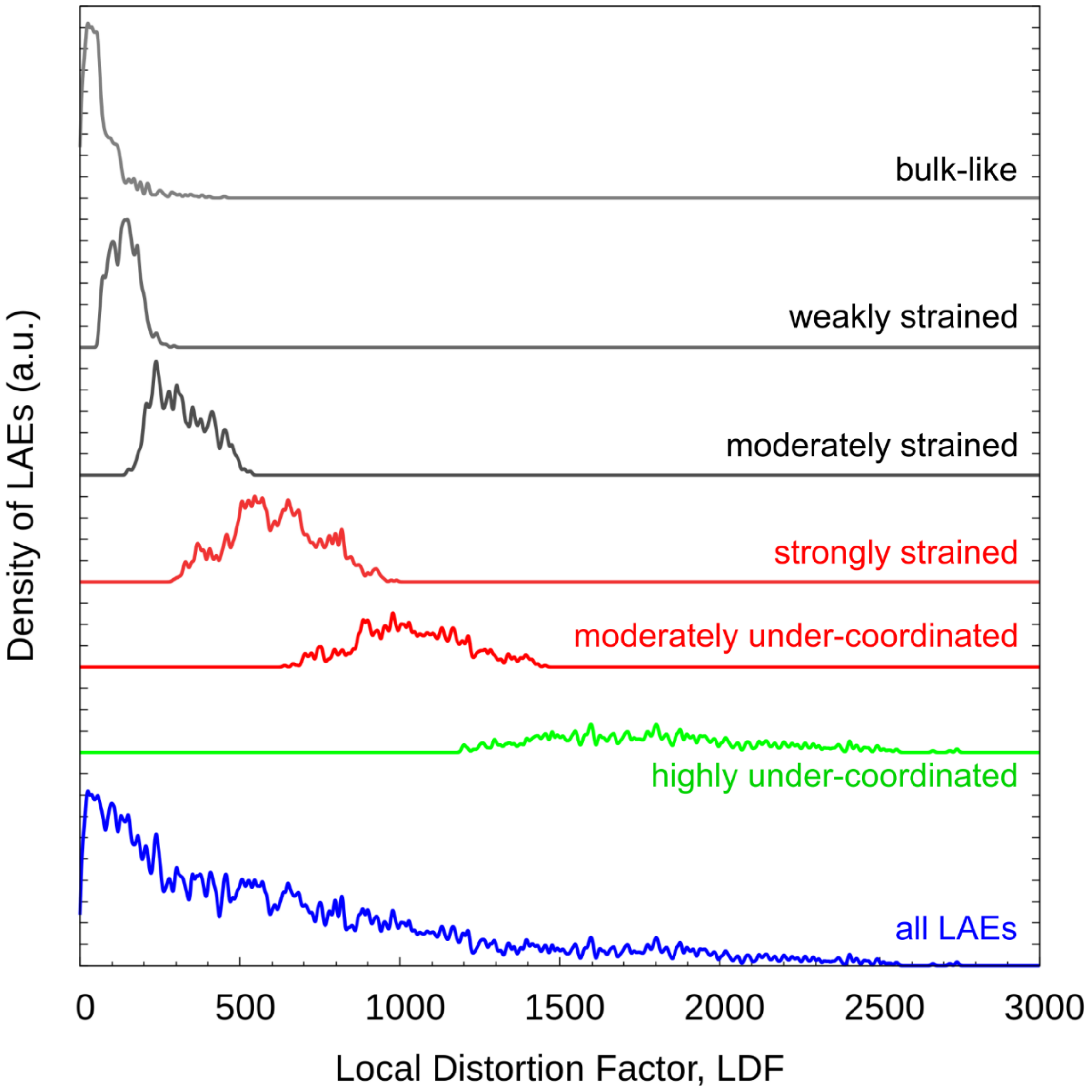

Supplementary Fig. 3 Density of LAEs of all grain boundary models as a function of local distortion factor, LDF, for six LAE groups, namely bulk-like, weakly strained, moderately strained, strongly strained, moderately under-coordinated, and highly under-coordinated. The bottommost plot labelled "all LAEs" is the summation of the densities of all six LAE groups.

Supplementary Note 2

The GB dataset used in this study contained many types of MgO GBs (low- and high-angle symmetric tilt GBs for six different rotation axes; twin and twin-like GBs; twist, high-pressure tilt and asymmetric tilt GBs), as listed in Supplementary Tables 1 to 9, and was thus very diverse in terms of crystallography, energetic stability and local number density of atoms at GB planes. Supplementary Fig. 2 shows plots of three macroscopic parameters reflecting this diversity: coincident site lattice density ($1/\Sigma$); GB energy and GB excess volume. The diversity in GB types ensures that the range of LAEs identified using hierarchical clustering is sufficiently broad to encompass those occurring in the majority of GBs likely to be encountered. Supplementary Fig. 3 shows this diversity in terms of LDFs for each group of the six LAEs, from bulk-like to highly under-coordinated. The robustness and transferability of the ML model is demonstrated by its ability to accurately predict GB thermal conductivities of asymmetric tilt GBs even though these data were not used as training data.

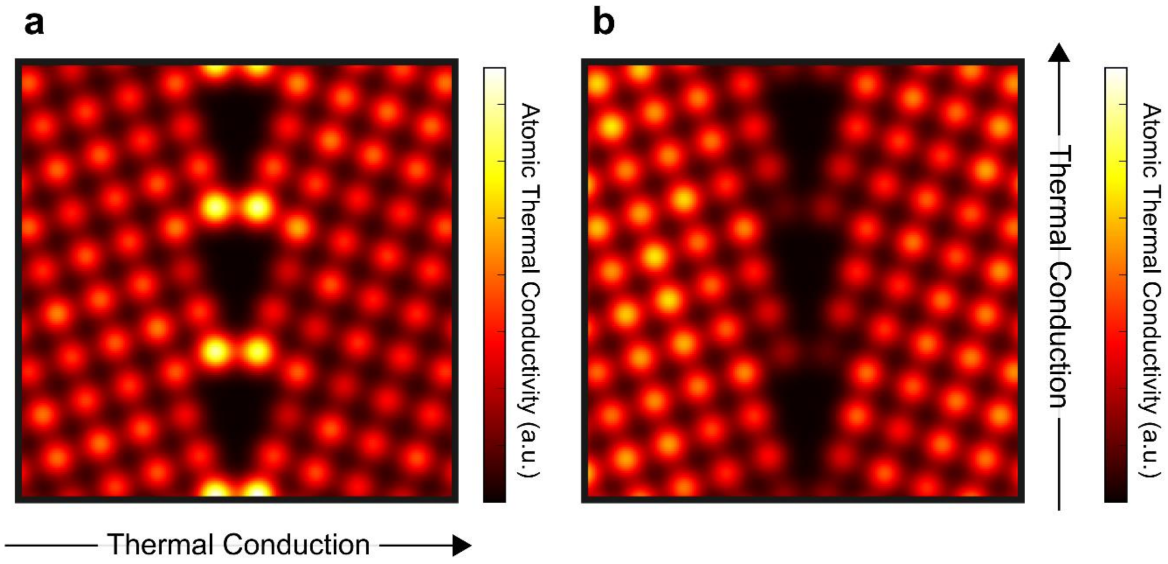

Supplementary Fig. 4 Gaussian smeared atomic thermal conductivities in two different directions at a Σ5(310)/[001] GB of MgO: **(a)** Perpendicular to the GB plane and **(b)** parallel to the GB plane. Thermal conductivities of atoms adjacent to the GB plane are highest across the plane but lowest along it, indicating a strong anisotropy in thermal conduction at this GB. Note that atomic thermal conductivities in **a** and **b** are normalized values.

Supplementary Note 3

Atomistic thermal conductivities at GBs can be highly anisotropic, as shown in Supplementary Fig. 4. The SOAP descriptor, however, is non-directional because it was developed to describe an atomic environment in a rotationally invariant form. This is not a serious issue when considering thermal conduction because phonons are mainly disturbed in the direction perpendicular to the GB plane, and the ML model can be made to "learn" from data pertaining to this direction. Even when chain-like atom linkages with high LDFs and high atomistic thermal conductivity occur, they are balanced by the lack of thermal conduction across the voids immediately adjacent to them. ML models for other properties, such as ionic diffusion, however, may suffer from this lack of directionality. One possible solution for this may be to add a metric describing the interconnectivity of LDF values in network form. For other systems, descriptors that include directionality implicitly, such as force-field-inspired descriptors [2] method may be more suitable.

Supplementary Note 4

To investigate whether LDF itself is a good descriptor for GB thermal conductivity, another ML model was constructed with LAE groups simply divided by LDF values, e.g., (1) $0 \leq \text{LDF} \leq 104.4$, (2) $104.4 < \text{LDF} \leq 227.8$, (3) $227.8 < \text{LDF} \leq 463.3$, (4) $463.3 < \text{LDF} \leq 821.1$, (5) $821.1 < \text{LDF} \leq 1409.2$ and (6) $1409.2 < \text{LDF}$, where the limits are the means of the average LDF values of two adjacent LAE groups classified using hierarchical clustering. This model also showed good predictive performance for GB thermal conductivity, with a root mean square error (RMSE) and R^2 value of 1.34 $Wm^{-1}K^{-1}$ and 0.92, respectively, for the training data, and 1.61 $Wm^{-1}K^{-1}$ and 0.89, respectively, for the test data. It was found, however, that in this case the regression coefficients of the LAE groups are very sensitive to the threshold values, making it difficult to interpret the thermal conduction mechanism in terms of LAEs. This is mainly because, using the mean-LDF method, similar LAEs are not identified as reliably as with Ward's hierarchical clustering method; in other words, LDFs provide a means of roughly estimating the magnitude of local structural distortion relative to the bulk crystal, and thus its likely effect on thermal conductivity, but as they contain no information about the nature of that distortion, the correlation between LDF and thermal conductivity is not as strong as that using groups identified using Ward's clustering method, at least for low LDF values. As shown in Supplementary Fig. 3, hierarchical clustering produces LAE groups in which most of the atoms have different LDF values to those in other groups, but there is some overlap between neighbouring groups, especially for the low LDF groups; these "intermediate" environments are thus difficult to classify reliably using simple LDF thresholds. Despite LDF alone not being ideal as a descriptor, however, with the help of hierarchical clustering of LAEs it is possible to construct a robust prediction model, and at the same time use LDFs to help interpret the physical mechanism behind thermal conductivity suppression.

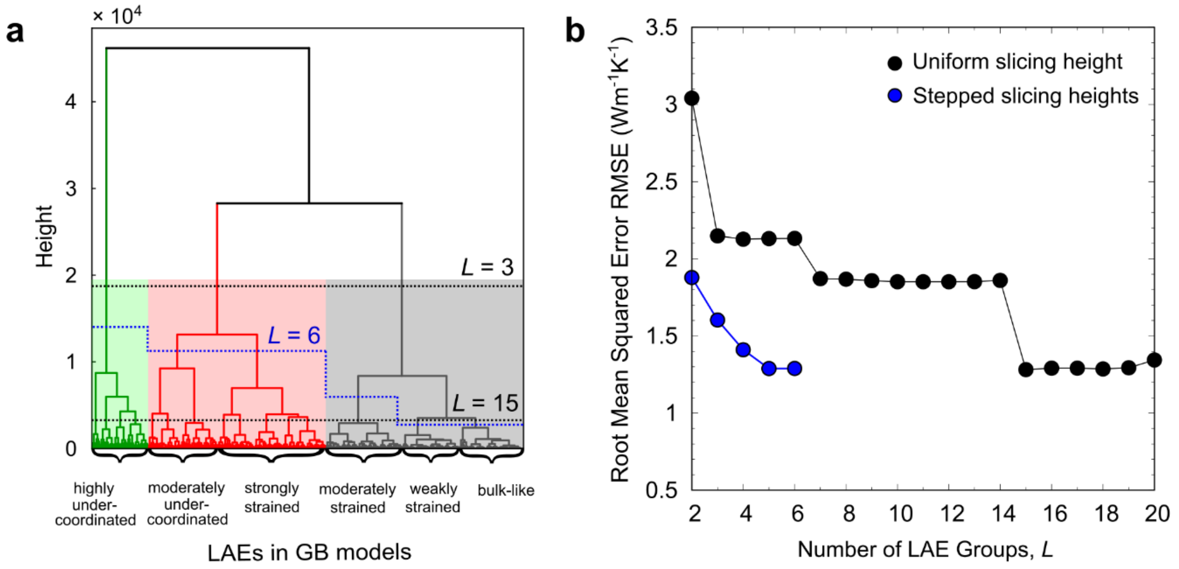

Supplementary Fig. 5 Dependence of predictive performance on number of LAE groups, *L*, used in prediction models for GB thermal conductivity. **a** Hierarchical relationship between LAE groups depicted in dendrogram form. The coloured regions represent three general groups of LAEs: (green) highly under-coordinated (bond-ruptured); (red) moderately under-coordinated or strongly strained; and (grey) moderately strained, weakly strained or bulk-like. **b** Root mean square error (RMSE) for all data (including both training and test data) as a function of *L*. LAE groups were categorized in two ways: 1) by slicing horizontally all branches at a particular height (black) manually slicing branches at different heights (blue). Example groupings for these two selection methods are indicated using dotted lines of the corresponding colour in **a**.

Supplementary Note 5

Supplementary Fig. 5b shows that RMSEs for the models using results of the hierarchical clustering method in Supplementary Fig. 5a decreased substantially when L was increased from 2 to 15 using a naïve slicing method (indicated by horizontal lines in Supplementary Fig. 5a). In this case, when $L < 15$, weakly strained and bulk-like LAEs are placed in a single group, whereas for $L \geq 15$ they are separated into two groups (described as "weakly strained LAEs" and "bulk-like LAEs"). This result shows that it is important to distinguish between bulk-like LAEs and more distorted LAEs in order to predict GB thermal conductivities accurately.

To confirm this, we also tested alternative grouping schemes by slicing branches manually, varying the slicing heights to incrementally increase L from 2 to 6 (blue dotted lines). Supplementary Fig. 5b shows that the RMSE values using this grouping method are much lower than those using the hierarchical grouping method for the same number of LAE groups, confirming the importance of distinguishing between bulk-like and slightly-distorted LAEs. This shows that care must thus be taken to ensure the number of groups selected is sufficient to incorporate all physically pertinent distinctions to obtain good predictive performance. Although RMSE converged with $L = 5$, in which highly and moderately under-coordinated atoms are placed in a single group, six LAE groups ($L = 6$) were chosen to provide a more robust understanding of the relationship between LAEs and GB thermal conductivities.

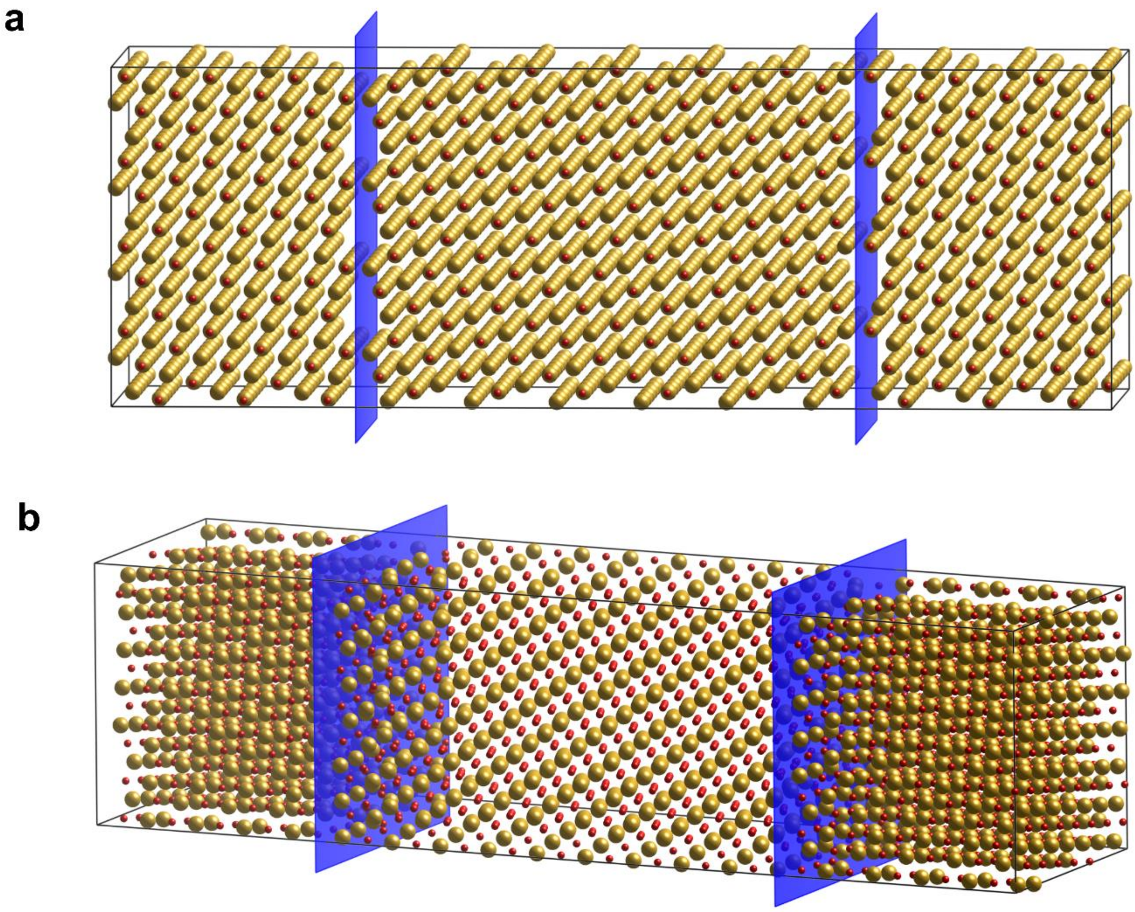

Supplementary Fig. 6 Examples of GB models used for thermal conductivity calculations and structure analysis. **a** Σ5(310)/[001] STGB; **b** Σ25(001) twist GB. Large yellow and small red balls represent Mg and O ions, respectively. Distances between GB planes (shown in blue) are about 4 nm in both cases.

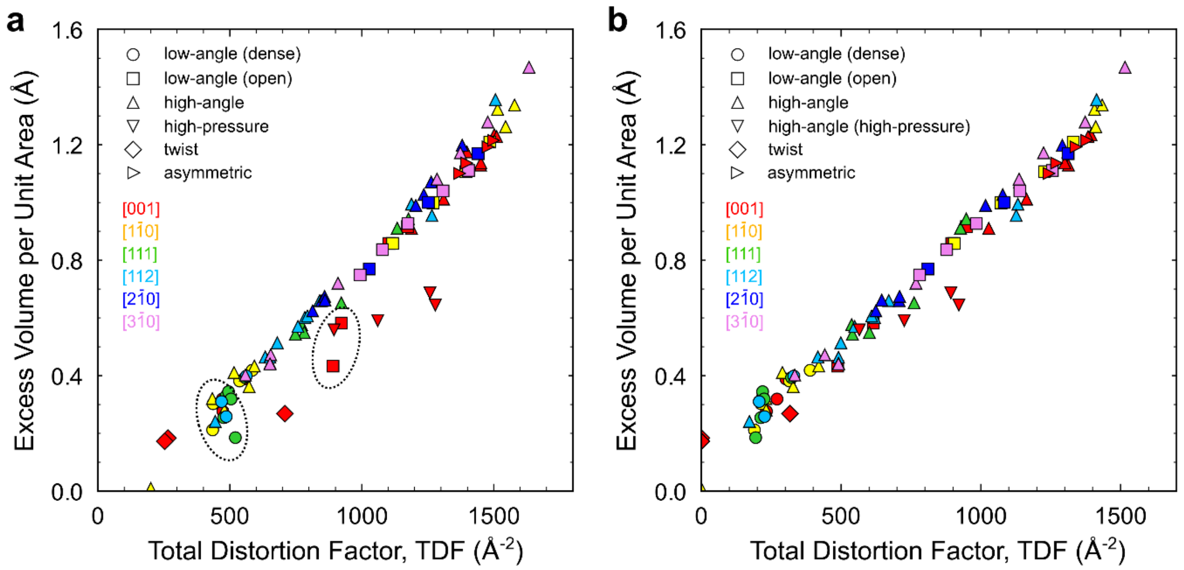

Supplementary Fig. 7 Excess volume per unit area as a function of total distortion factor, TDF. The TDF of each GB model was calculated by summing the local distortion factors, LDFs, of **(a)** all atoms in the GB model and **(b)** only atoms with strongly strained or under-coordinated atoms according to the hierarchical clustering in Supplementary Fig. 5a.

Supplementary Note 6

In Supplementary Fig. 7a, a linear relationship is evident between excess volume and TDF in the case of high-angle STGBs formed under standard pressure. This suggests that LDFs are related to the local excess volume near a GB core, at least in the case of MgO. This relationship is likely particularly strong in the case of MgO because the simple FCC symmetry of its sublattices typically produces well-ordered GB structures retaining many like-unlike nearest neighbour atom pairs and few like-like pairs. Bond angles at MgO GBs also tend not to be very different to those in the crystal bulk for the same reason. Compounds with more complex structures and atoms in lower coordination environments may exhibit more drastic changes in bond angles and LAEs at GBs than MgO, which could result in larger deviations from this linear relationship. In the case of low-angle tilt GBs (indicated by dashed circles in Supplementary Fig. 7a) deviation from the trend for high-angle STGBs implies atoms are subject to large elastic strain fields which do not produce as great an excess volume as the high-angle STGBs. When TDFs of each GB were re-calculated excluding atoms belonging to the "moderately strained, weakly strained and bulk-like" LAE group (Supplementary Fig. 5a), we found that the low-angle tilt STGBs fit to the same trend as the high-angle STGBs (Supplementary Fig. 7b). This difference in the correlation between excess volume and LDFs vis-a-vis thermal conductivity is consistent with the complex relationship in Fig. 1a of the main text compared to correlations based on the SOAP descriptor and LDFs.

Twist GBs and high-pressure STGBs also deviate from the main trend, but this is likely because the relationship between excess volume and TDF is different in these cases. More data for these GBs is needed to confirm this, however.

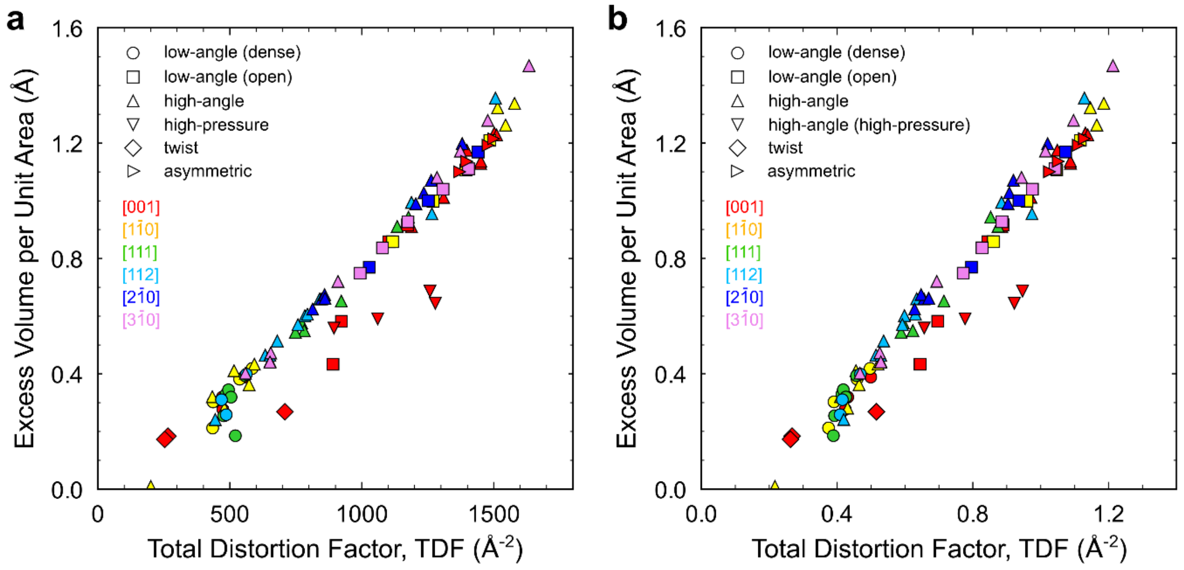

Supplementary Fig. 8 Excess volume per unit area as a function of total distortion factor, TDF, obtained using two computer programs: **(a)** DScribe [3] and **(b)** QUIP [4]. Both produced an almost identical linear relationship between excess volume and TDF. Note that a larger cutoff of 6.0 Å was needed for QUIP to obtain the same result as DScribe with a cutoff of 4.461 Å. This may be because of the different implementations of the cutoff region transition in the two programs, as suggested in the supplementary information of Jäger et al. [5].

Supplementary Methods

Analysis of the data was mostly performed by writing Python scripts in-house and utilizing DScribe [3], SciPy [6], scikit-learn [7] and Atomic Simulation Environment (ASE) [8] libraries, which are distributed under the Apache License version 2.0, SciPy's license, BSD license and GNU LGPL license, respectively. Here we explain the most salient points in constructing Python scripts to reproduce our results.

First, we calculated SOAP vectors of atoms in GB models using DScribe libraries in the manner:

```
from dscribe.descriptors import SOAP
from ase.io import read
soap = SOAP(species=["Mg", "O"], periodic=True, rcut=4.461, nmax=12, lmax=9, sparse=False)
atom = read(filename, format="lammps-data")
soaps_gb = soap.create(atom, n_jobs=1)
```

In the case of MgO, Mg and O atoms can be treated equivalently, with the same Gauss distribution widths, because their site symmetries are the same, both occupying FCC sublattices. This simplifies the machine learning model, and makes it easier to understand the relationship between LAEs and GB thermal conductivities in this material. However, in the DScribe code, by default components of the SOAP vector are listed in order of increasing atomic number of the interacting atoms, and thus the SOAP vectors for Mg and O atoms are not consistent, even in the case of a single crystal. In our analyses, in the lines of code following those above, we re-ordered the SOAP vector components (soap_gb) so that they are listed in the same order as the coordination shells, i.e., O-O interactions first, then Mg-O interactions, followed by Mg-Mg interactions in the case of Mg-centred SOAP vectors, and the reverse order for O-centred vectors.

Next, we generated a set of LAEs for each GB model using hierarchical clustering with the complete linkage algorithm using SciPy libraries as follows:

```
from scipy.cluster.hierarchy import linkage, cut_tree
result = linkage(soaps_gb, metric='euclidean', method='complete')
cutree = cut_tree(result, height=30.0)
```

The geometric centre, or centroid, of the SOAP vectors in each LAE set was calculated, and the atom with the closest SOAP vector to the centroid was considered to be representative of that LAE.

Next, SOAP vectors of all LAEs in all GB models were placed in a single list, soaps_LAEs, and Ward's method of hierarchical clustering was performed to classify the LAEs into groups:

```
from scipy.cluster.hierarchy import linkage, plot_dendrogram
result = linkage(soaps_LAEs, metric='euclidean', method='ward')
plot_dendrogram(result, "dendrogram.eps")
```

The LAE groups were determined based on the outputted dendrogram. Atoms and their coordinates in the groups were then listed for each GB model, and the number of LAEs per unit area of a GB, N_m, calculated according to Equation (8) in the main text.

Finally, multiple linear regression (ridge regression) was performed using scikit-learn libraries for the leave-one-out cross-validation method:

```
from pandas as pd
from sklearn.linear_model import RidgeCV
from sklearn.model_selection import train_test_split

df = pd.read_csv(datafile, "sep="¥s+", header=None)
train, test = train_test_split(df, test_size=0.2, random_state=seed)
asym = pd.read_csv(datafile_asymmetric, sep="¥s+", header=None)
test = pd.concat([test, asym])
…
clf = RidgeCV(alphas=[1.0e-8, … , 1.0e-1], cv=None, fit_intercept=False)
clf.fit(train_n, train_k)
```

The impact of each LAE group on GB thermal conductivity was then estimated in terms of the regression coefficients.

Supplementary References

[1] Fujii, S., Yokoi, T. & Yoshiya, M. Atomistic mechanisms of thermal transport across symmetric tilt grain boundaries in MgO. *Acta Mater.* **171**, 154–162 (2019).

[2] Choudhary, K., DeCost, B. & Tavazza, F. Machine learning with force-field-inspired descriptors for materials: Fast screening and mapping energy landscape. *Phys. Rev. Materials* **2**, 083801 (2018).

[3] Himanen, L. *et al.* DScribe: Library of Descriptors for Machine Learning in Materials Science. *Comput. Phys. Commun.* 106949 (2019).

[4] Bartók, A. P., Payne, M. C., Kondor, R. & Csányi, G. Gaussian approximation potentials: The accuracy of quantum mechanics, without the electrons. *Phys. Rev. Lett.* **104**, 1–4 (2010).

[5] Jäger, M. O. J., Morooka, E. V., Federici Canova, F., Himanen, L. & Foster, A. S. Machine learning hydrogen adsorption on nanoclusters through structural descriptors. *npj Comput. Mater.* **4**, 37 (2018).

[6] Jones, E., Oliphant, T., Peterson, P. *et al.* SciPy: Open Source Scientific Tools for Python, http://www.scipy.org/ (2001-).

[7] Pedregosa, F. *et al.* Scikit-learn: Machine Learning in Python, *J. Mach. Learn. Res.* **12**, 2825-2830 (2011).

[8] Hjorth Larsen, A. *et al.* The atomic simulation environment **-** A Python library for working with atoms. *J. Phys. Condens. Matter* **29**, 273002 (2017).
