# Supplementary material for: Quantitative prediction of grain boundary thermal conductivities from local atomic environments
Source: Nat Commun. 2020 Apr 15;11:1854. doi: 10.1038/s41467-020-15619-9 (PMC7160158; doi:10.1038/s41467-020-15619-9)
Supplement: Supplementary file 3 — Description of Additional Supplementary Files [file 41467_2020_15619_MOESM3_ESM.pdf]

Description of Additional Supplementary Files

File name: Supplementary Data 1

Description: GB models used in this study in LAMMPS format
